# Innate Immunity and Influenza A Virus Pathogenesis: Lessons for COVID-19

**DOI:** 10.3389/fcimb.2020.563850

**Published:** 2020-10-22

**Authors:** Kevan L. Hartshorn

**Affiliations:** Section of Hematology Oncology, Boston University School of Medicine, Boston, MA, United States

**Keywords:** influenza, neutrophil, surfactant protein D, LL-37, cytokine storm, pandemic, defensin, SARS-CoV

## Abstract

There is abundant evidence that the innate immune response to influenza A virus (IAV) is highly complex and plays a key role in protection against IAV induced infection and illness. Unfortunately it also clear that aspects of innate immunity can lead to severe morbidity or mortality from IAV, including inflammatory lung injury, bacterial superinfection, and exacerbation of reactive airways disease. We review broadly the virus and host factors that result in adverse outcomes from IAV and show evidence that inflammatory responses can become damaging even apart from changes in viral replication *per se*, with special focus on the positive and adverse effects of neutrophils and monocytes. We then evaluate in detail the role of soluble innate inhibitors including surfactant protein D and antimicrobial peptides that have a potential dual capacity for down-regulating viral replication and also inhibiting excessive inflammatory responses and how these innate host factors could possibly be harnessed to treat IAV infection. Where appropriate we draw comparisons and contrasts the SARS-CoV viruses and IAV in an effort to point out where the extensive knowledge existing regarding severe IAV infection could help guide research into severe COVID 19 illness or vice versa.

## Introduction

There is a vast, and still growing, literature on the pathogenesis of severe IAV infection. A major concern has been adaptation of avian viral strains [e.g., those bearing different hemagglutinin (HA) molecules like H5, H7, or H9] to human to human transmission. There is a strong system of surveillance for human infection with such avian strains that has been effective in containing outbreaks in domestic animals to lower the risk for human transmission. The last IAV pandemic (2009) was not caused by a virus bearing a novel HA but the re-emergence from a porcine source of an H1N1 strain similar to the 1918 pandemic IAV. The current catastrophic SARS-CoV2 pandemic (and related SARS-CoV1 and MERS outbreaks) emerged from different sources altogether. This does not lessen the need for avian and porcine IAV surveillance. However, it is reasonable to consider what we know about severe IAV pathogenesis for lessens applicable to other severe respiratory virus infections, like COVID 19, to seek common points that could be used to guide treatments. In both cases it appears that innate immunity plays a central role in early protection but also in the most adverse outcomes.

Based on the high degree of variability of IAV strains from year to year through antigenic drift and the potential for reshuffling of whole genome segments through reassortment, adaptive immune responses generated against prior strains (or prior vaccines) can be of limited or possibly no effectiveness vs. a new strain. Coronaviruses may not have as high a propensity to variation and do not have a multi-segmented genome as does IAV. However, there are many strains of coronavirus in animal reservoirs so that novel infections with these strains are a major risk. Whereas, seasonal IAV tends to be somewhat attenuated due to adaptations made for sustained maintenance in humans and due to partial immunity against them, highly pathogenic coronavirus infections resemble more closely novel pandemic IAV strains in that humans are immunologically largely naïve to them. These factors leave innate immunity with a key role in bridging the gap between initial infection and onset of cell and antibody mediated immunity for both IAV and novel coronaviruses.

The overall topic of innate immunity to IAV is quite vast we will not be able to review it in great depth but rather will refer to recent reviews for more detail. We will summarize viral and host factors established to be involved in the development of severe IAV infection. We will then explore in more depth evidence for a role for myeloid cells in host defense and damaging inflammation during IAV infection. We will then summarize the work of our and other labs on the role of key soluble innate inhibitors including surfactant protein D (SP-D) and antimicrobial peptides (defensins and LL-37) in host defense and potential treatment of IAV infection. Where appropriate draw comparisons or contrasts to COVID-19, since knowledge of severe IAV pathogenesis may allow a more rapid discovery of aspects of pathogenesis of severe COVID 19 and conversely treatments being tested for COVID-19 may prove beneficial for severe IAV as well (see Table 1).

**Table 1:** Similarities and differences between IAV and SARS-CoV2 infection.

**Feature**	**IAV**	**SARS-CoV2**
Receptor	α 2,6 sialic acids (human) α 2,3 sialic acids (avian)	ACE2
Genome	Multisegmented RNA	Single RNA Segment
Mechanisms of Genome Variation	Reassortment Point mutation	Point mutation
Morbidity and Mortality	Bacterial Superinfection>Viral Pneumonia	Viral Pneumonia, ?Bacterial Superinfection Cytokine storm with IL-6 Thrombosis
Current Antiviral Therapy	M protein inhibitors, neuraminidase inhibitors	Remdesivir
Emerging Therapy	Favipiravir	Interferons Favipiravir
Increased Susceptibility for Older Adults	Yes (unless previously immune)	Yes
Co-morbidities Related to Severe Illness	Cardiovascular Disease, Obesity, Diabetes, COPD	Cardiovascular Disease, Obesity, Diabetes, COPD
Anti-inflammatory Therapy	None approved	Dexamethasone ? Cytokine blockers
Animal Reservoirs	Birds, Pigs	Bats, ?Felines ?Pangolins
Binding protein	HA	S protein
Binding protein glycosylation	High in seasonal, human adapted strains Low in pandemic and avian strains	High
Cell Targets	Respiratory Epithelial Cells	Respiratory Epithelial Cells, Endothelial Cells, ?others with ACE2 expression
Neutrophilic infiltration	Yes—early influx beneficial late influx maybe harmful	Yes—role not yet evaluated but may be similar
Inflammasome activation	Yes	Likely (confirmed for SARS-CoV1)
Type III IFN production	Contributes to host defense and bacterial superinfection	Contributes to host defense and bacterial superinfection
Viral proteins to block IFN action	Yes	Yes
Elevated CRP and SAA	Yes	Yes
Role of SP-D	Antiviral and anti-inflammatory (seasonal glycosylated strains inhibited)	Unknown
Role of antimicrobial peptides	Antiviral and anti-inflammatory	Unknown

? *Indicates possible features of SARS-CoV infection pending further study*.

### Broad Overview of Innate Immunity to IAV

**Figure 1:**
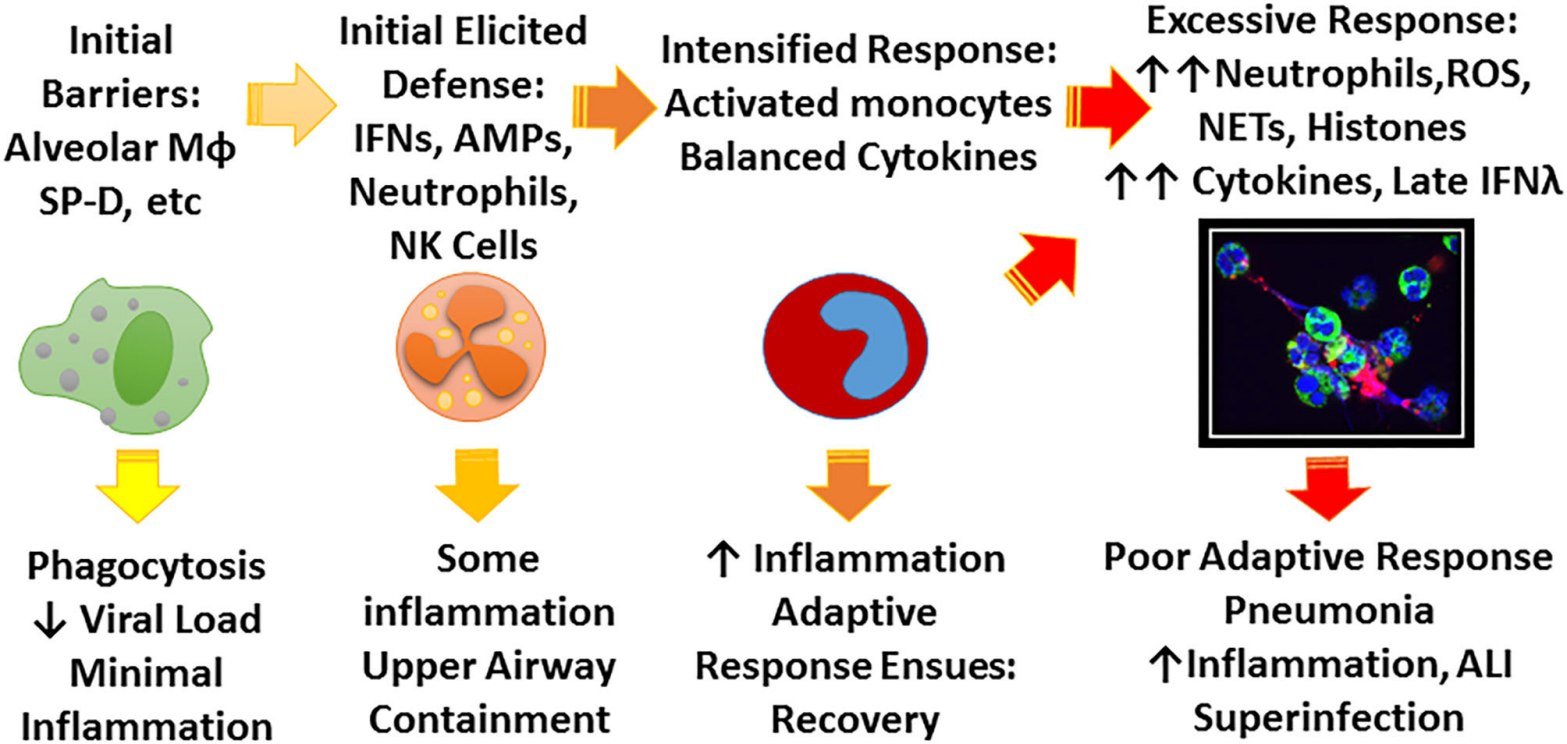
Schema for possible stages of IAV infection. IAV initially encounters a number of constitutive barriers to infection, followed by locally elicited responses which help contain the virus, prevent spread to the lung, and begin to trigger adaptive immune responses. The principle role of the early innate response is to limit viral replication and minimize inflammation until adaptive response ensues. Depending on characteristics of the viral strain (e.g., pandemic vs. seasonal, HA characteristics) and host factors (e.g., immune deficiency, underlying inflammatory bias) the infection may not be well-contained leading to more inflammation and ultimately lung infection which can lead to profound illness through excessive inflammatory responses and possibly bacterial superinfection. The figure highlights the role of soluble inhibitors, monocyte/macrophages, and neutrophils which are emphasized in this review. The confocal microscope picture shows neutrophils treated *in vitro* with IAV and forming NETS. Green stain highlights neutrophil plasma membranes, blue stain highlights nuclei and cell free DNA, and red stain indicates virus.

There is an extensive literature on the extraordinary innate immune response to IAV which plays a key role as a bridge between initial infection and onset of adaptive immunity (Tripathi et al., 2015c; Biondo et al., 2019). Ultimate viral clearance depends on adaptive immunity including generation of an effective CD8 response and neutralizing antibody responses. These adaptive responses are key for protection against re-infection but, due to the variable nature of IAV, protection is not long lasting. However, an amazing demonstration of the long lasting nature of adaptive responses is the finding that people who survived the 1918 pandemic still retained protective antibodies against the 2009 H1N1 which was closely related to 1918 strains (and had survived porcine sources long enough to resurface) (Xu et al., 2010). Innate immunity can accelerate and inform the adaptive response and potentiate responses to vaccines, however, our focus will be mainly on initial innate defense where the battle is often lost or won. The key to effective early innate responses is to limit viral replication at least partially without causing inflammatory damage. Figure 1 shows a schema of stages of response to IAV that we will focus on in this review.

We propose that the first line of defense includes soluble factors in the respiratory tract, the respiratory epithelium, and resident pulmonary immune cells (alveolar macrophages). Of interest some soluble factors like the collectins (including SP-D, surfactant protein A or mannose binding lectin), LL-37 and also alveolar macrophages appear to limit viral replication while also inhibiting inflammation (LeVine et al., 2001, 2002; Barlow et al., 2011; Nelson et al., 2014). A key outcome can be restriction of virus replication to upper airways and prevention of lung infection. The next phase of response involves generation of interferons locally (including IFNlambda produced by respiratory epithelial cells) (Wang et al., 2009) and chemokines which result in recruitment of other immune cells, notably NK cells, neutrophils, recruited monocyte (in contrast to resident AMs) and innate lymphocytes. NK cells can kill virus infected cells and also produce IFN gamma. Dendritic cells start to play a key role in triggering adaptive responses and releasing Type 1 IFNs.

In the case of IAV, a key turning point can be viral infection of the lung epithelium (e.g., penetration beyond upper airway barriers). If viral infection does reach the lung profound illness can occur and can be associated with severe outcomes. Of note, SARS-CoV2 appears to have greater potential to achieve lung infection (perhaps due to greater binding affinity for ACE2 receptors present in the lung), whereas IAV has highest affinity for upper airway epithelia which contain alpha-2,6 linked sialic acids needed for hemagglutinin (HA) binding (Imai and Kawaoka, 2011). In addition SARS-CoV2 appears to infect endothelial cells and thrombosis and other vascular effects are coming to the fore as important mediators of morbidity in COVID-19 infection (Ackermann et al., 2020; Cure and Cure, 2020; Sardu et al., 2020). H5N1 IAV infection is associated with much higher mortality in humans in part because of its affinity for alpha-2,3 sialic receptors in the distal lung epithelium leading to lung infection and profound inflammation that has been termed a “cytokine storm” (a term now used often in discussing COVID 19). If lung infection with human IAV strains happens before adaptive immunity can come to the rescue then profound inflammation can occur. It is unclear if a truly damaging inflammatory response can occur outside of lung infection in otherwise healthy people. However, lower airway infection and cytokine responses may be sufficient to lead to severe illness in patients with underlying lung or cardiovascular disease through causing respiratory failure or cardiac/thrombotic events (Nichol et al., 2003). Widespread lung inflammation can impair oxygen exchange and result in damage to the epithelial capillary barrier.

Major pro-inflammatory cytokines like TNF and IL6 drive symptoms as does type I IFN when systemically produced, but also result in massive recruitment of neutrophils and other inflammatory cells to the lung (Hayden et al., 1998; Hagau et al., 2010; Gong et al., 2011; Paquette et al., 2012). Recruited monocytic cells can be factories for production of these cytokines through RIG-1/MAVS, TLR and inflammasome pathways and may be a central player in the “cytokine storm.” Similar pathways are likely at work during COVID-19 leading to trials of treatment with anti-inflammatory drugs. Our major focus for this review will be on severe IAV such as is seen during pandemic infection or avian influenza infection (or most murine models of infection), and we will focus primarily on specific aspects of the immune response that we have studied that may relate to future treatments.

### Viral Factors Leading to Severe IAV Infection

Pandemic human strains and avian strains which have infected humans through direct animal to human contact cause high levels of inflammation and increased mortality in humans. The lung pathology in these cases includes massive neutrophil and monocyte influx (Perrone et al., 2008). As noted in the case of the 1918 pandemic a high proportion of autopsy cases showed bacterial superinfection as well (Morens et al., 2008). The role of bacterial superinfection is less clear in COVID-19 but recent data suggests it may be an important factor at least in ventilated patients (see below).

**Figure 2:**
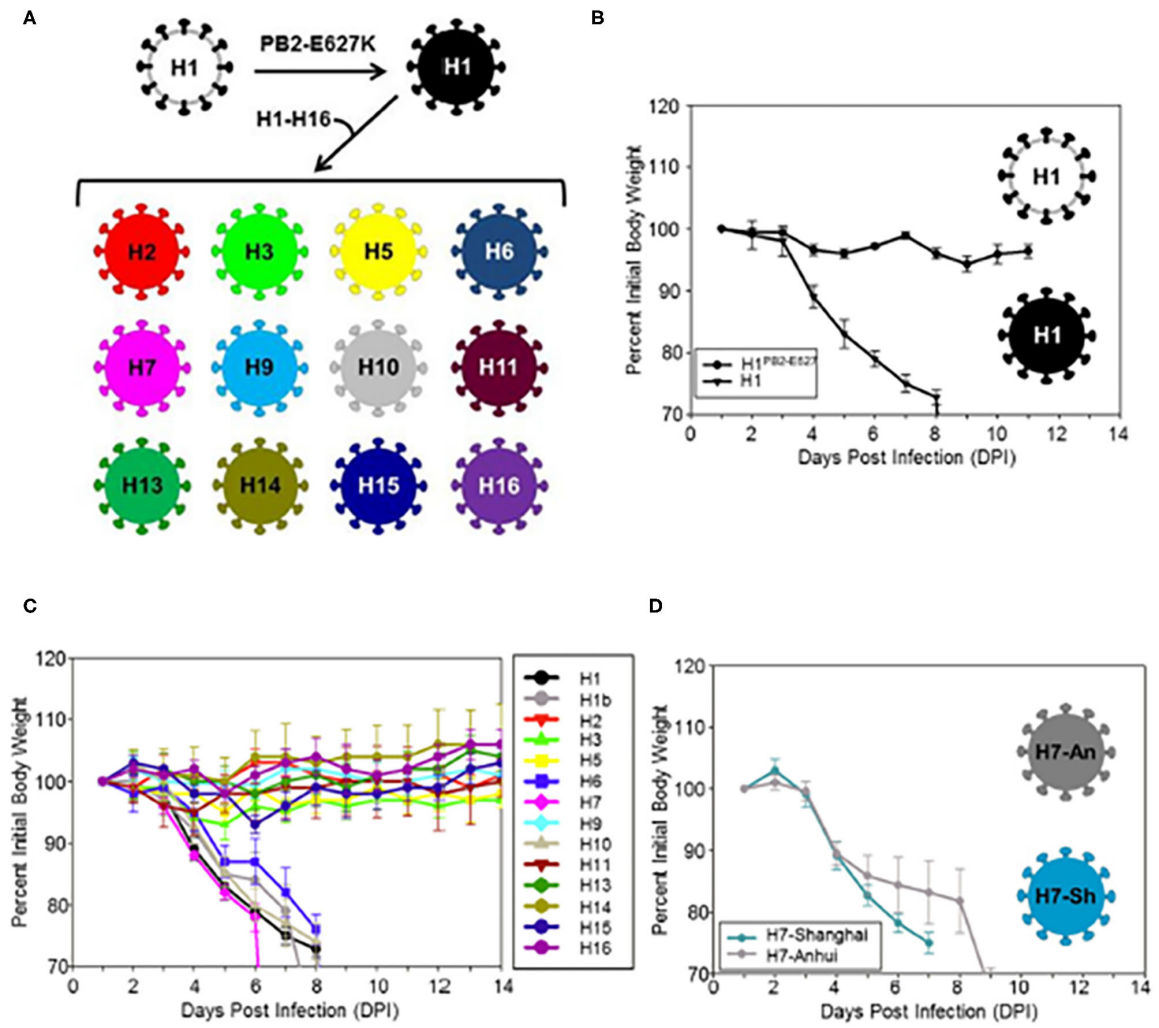
**(A)** Illustrates construction of viruses differing from H1 (black colored) only is thus HA molecules. **(B–D)** Analysis of weight loss in mice infected with modified avian strains differing only in their HA molecules. Note that viruses contained the H1PB2-627E, H6, H7, H10, and H15 HA caused massive weight loss. This was accompanied by loss of resident macrophages and marked influx of neutrophils and inflammatory macrophages, pathogenic effects on human bronchial epithelial cells in culture and ultimate mortality. Viruses containing H2, H3, H5, H9, H11, H13, H14, and H16 HA were not pathogenic. Note that the pathogenic H1 virus was modified to contain a PB2 E627K mutation which is needed for mouse adaptation. Two different H7 strains were tested and they were similarly pathogenic. Only the H3 strain was inhibited by SP-D *in vitro* in this paper. These experiments implicate that IAV HA from different avian subtypes contains important virulence factors apart from other viral genes and that the HA can drive inflammatory myeloid cell infiltration. This figure was obtained and adapted (Qi et al., 2014). Use is allowed if properly cited.

The viral HA is a key factor in pathogenicity. This has been demonstrated with the reconstructed 1918 H1N1 strain. Reverse genetics has provided a remarkably effective tool in dissecting out the role of segments of IAV strains to pathogenesis. For instance strains constructed using only the HA segment of the 1918 H1N1 virus combined with segments of a lower pathogenicity seasonal virus produce fatal infection in animal models very similar to the intact 1918 strain, including massive lung damage and infiltration with neutrophils and other myeloid cells (Kobasa et al., 2004). Similar experiments showed that in addition to the HA, the NA and PB1 genes of the 1918 virus contributed to high virulence (Kobasa et al., 2007). There is evidence that reactive oxygen and nitrogen species contribute to lung damage based on prior studies (Tripathi et al., 2015c). A recent study showed that HA molecules of H1, H6, H7, H10, and H15 of avian strains confer a similar severe inflammatory phenotype, characterized by massive cytokine and chemokine expression, strong upregulation of reactive oxygen, and nitrogen responses, and strong inflammatory and cell death signatures (Qi et al., 2014; Figure 2). Cellular infiltrates in the lung were predominantly composed of neutrophils and inflammatory macrophages (with loss of resident alveolar macrophages). In contrast strains containing HA genes of H2, H3, H9, H11, H13, and H16 caused mild weight loss and no fatalities, despite having the same set of the seven other genes as the H1, H6, H7, and H15 strains which did cause a high level of pathogenicity. These studies indicate that there is an intrinsic virulence factor present in certain avian HA subtypes.

For a virus with a relatively small complement of genes IAV dedicates at least 4 genes to modulating host defense responses. The IAV NS1 protein is a remarkable multi-tasker and it counteracts IFN responses at multiple levels (see Tripathi et al., 2015c for review). The NS1 protein may account for the increased pathogenicity of H5N1 but not necessarily for that of 1918 pandemic H1N1 (Imai et al., 2010; Ma et al., 2010). IAV lacking NS1 is greatly attenuated and it is necessary to propagate the virus in NS1 expressing cells to attain usable viral titers. Other recently identified IAV proteins include PB1-F2, PA-X, and H5N1 microRNA. The PB1-F2 is derived from an alternative reading frame of the viral PB polymerase protein and is causes monocyte apoptosis and promotes bacterial superinfection among other effects (Conenello et al., 2007; McAuley et al., 2007; Mitzner et al., 2009). The PA-X protein in contrast actually acts to reduce inflammatory responses and deletion of it from the 1918 strain worsened infection outcomes (Jagger et al., 2012; Shi et al., 2012). The H5N1 microRNA increases cytokine production and mouse mortality by blocking PCBP2 which is a suppressor of RIG-1/MAVS (Li et al., 2018b). SARS-CoV and other respiratory viruses have similar non-structural proteins involved in modulating interferon and other innate host responses (Liu et al., 2014; Bengoechea and Bamford, 2020).

### Host Factors Leading to Severe IAV Infection

We cannot do justice to the vast literature on host factors linked to severe IAV infection in this review, but after a brief summary will focus in depth on some specific respiratory proteins that we have studied. Please see the following reviews for more general overviews of host factors involved in response to IAV (Tripathi et al., 2015c; Short et al., 2018).

#### Epidemiological Risk Factors

It is well-established that certain groups of people are at greatest risk during both seasonal epidemics and pandemics of IAV. These include smokers and others with lung disease, those with diabetes mellitus, pregnancy, obesity, cardiovascular disease, immune deficits, old age (except for subjects with prior immunity), and very young age. Malnutrition and co-infection with TB or malaria have also been noted as risk factors (Short et al., 2018). Many of these risk factors related to impaired adaptive immunity; however, others could relate to impaired or dysregulated innate immunity. For instance although obesity can adversely impact adaptive immunity it can also be linked to an underlying inflammatory bias that may predispose to excessive inflammatory response to infection (Hagau et al., 2010; Almond et al., 2013). Similarly old age is associated both with impaired adaptive immunity and, in some subjects, an underlying inflammatory state (termed “inflammaging” by some) (Elias et al., 2017; Chen et al., 2020). These two features may be linked. For instance, aged subjects have an increased tendency to accumulate myeloid derived suppressor cells that blunt adaptive immune responses (Chen et al., 2015). Diabetes mellitus is associated with impaired phagocyte function and adaptive immunity (Engelich et al., 2001). Elevated glucose can impair binding and function of SP-D as well, as demonstrated in mouse models (Reading et al., 1998). Underlying cardiovascular disease and “metabolic syndrome” are associated with an inflammatory phenotype as well. An important link exists between inflammation and coagulation and this may explain the increased risk for thrombotic events associated with severe IAV (Antoniak et al., 2013; Khoufache et al., 2013). Clearly further research on how known risk factors for severe IAV infection impact innate immunity is an important direction for future research. Severe COVID 19 appears to share a number of the same risk factors, including cardiovascular disease, COPD, diabetes, obesity, and old age (Chen et al., 2020). There is a striking association of severe COVID 19 illness with thrombosis which may have similar underpinnings as thrombosis occurring with severe IAV. However, COVID-19 appears to have a distinctive property (as compared to IAV) of infecting and damaging endothelial cells directly (Ackermann et al., 2020; Cure and Cure, 2020). This feature was recently found to distinguish lung pathology of fatal COVID-19 from fatal IAV pneumonia (Ackermann et al., 2020).

#### Genetic Risk Factors

Another challenge has been to identify why some otherwise healthy people succumb to severe IAV infection even during seasonal outbreaks. Based on recent studies several defects in innate immune response have been linked to severe IAV, including polymorphisms of interferon regulatory factors (IRFs) 7 and 9, interferon induced transmembrane protein (IFTIM3), complement component 1q binding protein and IL-1 among others (Zuniga et al., 2012; Tripathi et al., 2015c). Polymorphisms of SP-D exist which cause diminished ability to form high molecular weight multimers and these have reduced anti-IAV activity (Hartshorn et al., 2007). Polymorphisms of SP-D, SP-A and MBL have been linked to increased risk for respiratory viral infections including SARS-CoV1 (Zhang et al., 2005; Thomas et al., 2009a; Herrera-Ramos et al., 2014).

### Evidence That Myeloid Cells Can Be Both Helpful and Harmful in IAV Infection

There is abundant evidence that myeloid cells, including macrophages, monocytes and neutrophils, play important protective roles in IAV infection. Alveolar macrophages are key to initiation of a protective immune response, and play a strong protective role during IAV infection (Watanabe et al., 2005; Hashimoto et al., 2007; Kim et al., 2008; Tate et al., 2010; Wang et al., 2012). GMCSF can increase recovery from IAV via its promoting effects on macrophages (Huang et al., 2011; Sever-Chroneos et al., 2011). An important distinction can be made between resident macrophages and recruited monocytes which appears to be more harmful in IAV infection (see below). Neutrophils are the first cells recruited in abundance to the IAV infected airway and appear to play an important role in initial viral containment and facilitating the next levels of immune response. Neutrophils can promote adaptive response development and also limit viral replication and more severe inflammation in some studies (Tate et al., 2009, 2011a, 2012; Hufford et al., 2012). Neutrophils can take up IAV and aid in clearance of the virus (White et al., 2007). Even during severe IAV the profound blockade of myeloid cell infiltrates *per se* not protective and actually increased mortality in mice infected with 1918 H1N1 IAV (Tumpey et al., 2005).

The ability of the host cell to undergo autophagy and apoptosis in response to IAV is another key element of innate defense and cell death pathways is another major element of limiting viral spread and inflammation (Tripathi et al., 2015c). IAV accelerates neutrophil and macrophage apoptosis which would be expected to have an overall anti-inflammatory effect. The potential downside of this apoptosis is rendering the host susceptible to bacterial superinfection, which is a major complication of IAV infection (Rynda-Apple et al., 2015). IAV has been shown to trigger inflammasome activation leading to production of IL-1 by macrophages (Thomas et al., 2009b; Ichinohe et al., 2010; Pang and Iwasaki, 2011). A recent paper demonstrated that caspase 6 activation is important for regulating cell death responses and inflammasome activation in response to IAV (Zheng et al., 2020). In mouse models at least inflammasome activation and or IL-1 have been found to be protective vs. IAV (Schmitz et al., 2005; Belisle et al., 2010). A recent review of the potential role of inflammasome activation in COVID-19 evaluates the pros and cons of this response in severe coronavirus and IAV infections (Yap et al., 2020). In particular NLRP3 activation in alveolar macrophages or respiratory epithelia, which is linked to caspase-1 activation, production of IL-1 and IL-18, and pyroptosis, may cause harmful inflammation including excess cytokine production and neutrophil infiltration. They note that mechanical stretch as occurring in patients on ventilators exacerbates alveolar macrophage NLRP3 activation (Wu et al., 2013). The authors review some of the proposed treatments for modulating inflammasome activation noting also the contrary evidence for a beneficial role of NLRP3 activation in recovery from IAV infection.

There is also extensive evidence that myeloid cells contribute to damaging inflammation in the specific setting of severe IAV in some cases without improving control of the virus. Excessive neutrophil infiltration was found to underlie the increased mortality from IAV in older adults (Kulkarni et al., 2019; Chen et al., 2020). In contrast to alveolar macrophages, recruited monocytes do not necessarily mediate dampening effects on inflammation and can do the opposite (Lin et al., 2008, 2011). There are many examples from mouse models of IAV infection that viral replication can be uncoupled from inflammatory damage and related weight loss and death. Some of these are noted in our prior review (Tripathi et al., 2015c), including inhibition of early IL-17 responses (Crowe et al., 2009; Li et al., 2012a) and blockade of influx of activated monocytes in the lungs of mice infected with IAV (Lin et al., 2011). Both of these maneuvers had marked protective effects by lessening inflammation and improving survival without altering viral loads (Lin et al., 2011). Similarly, deletion of genes for CXCR3 or CXCL10 (otherwise known as IP-10 or IFN gamma inducible protein) led to improved survival with severe IAV infection through reduction of inflammation specifically including neutrophil recruitment, while not altering viral loads (Ichikawa et al., 2012). Note that CXCL10 is also highly elevated in SARS-CoV1 (Ichikawa et al., 2012). A very nice recent example is discovery of a microRNA encoded by H5N1 strains (and conserved in 455 different strains) that inhibits Poly(rC)-binding protein (PCBP2) which is a negative regulator of RIG-1/MAVS. RIG-1/MAVS is a major pathway through which IAV triggers type I IFNs and cytokines. Deletion of this sequence from the virus was achieved without altering viral replication *in vitro* or in mice, while markedly reducing mouse weight loss and mortality (Li et al., 2018b). In this study macrophages were the key source of cytokines driving inflammation. It should be noted that there are important distinctions between different subtypes or strains of IAV in their interactions with myeloid cells. For instance, avian H5N1 which causes a high rate of viral pneumonia and mortality in mice and humans has genetic differences in its internal proteins which allow for high level replication in myeloid cells leading to severe outcomes (Li et al., 2018a).

Interferons (IFNs) play fundamental roles in host defense against IAV and other viruses, as evidenced by the evolution of viral proteins (like the IAV NS1 and similar proteins for SARS-CoV1) (Ruckle et al., 2012). Type III IFN (IFN lambda) is being evaluated as a treatment for SARS-CoV2 (O'Brien et al., 2020; Prokunina-Olsson et al., 2020). IFN also exert regulatory effects on inflammasomes (Yap et al., 2020). Although IFNs play a key role in limiting viral replication, there is also evidence that they can have harmful effects. There is a strong interplay between IFNs and myeloid cells. An important study involving a mouse model of SARS-CoV1 found mice with knockout of the Type 1 IFN receptor or mice in which infiltrating inflammatory macrophages had markedly reduced inflammation and markedly improved survival, without significant change in viral titers (Channappanavar et al., 2016). Both Type 1 (alpha and beta) and Type II (gamma) IFNs have been found to contribute to the risk of bacterial superinfection which is a major cause of morbidity and mortality in IAV epidemics and pandemics (Sun and Metzger, 2008; Shahangian et al., 2009; Li et al., 2012b; Lee et al., 2015). These effects were mediated by IFN induced impairments of neutrophil recruitment and macrophage function. It is not fully clear yet the extent to which these similar factors result in bacterial superinfection in severe COVID-19 patients. Although not highlighted in initial studies it appears that bacterial superinfections due occur in COVID-19 (Bengoechea and Bamford, 2020; Cucchiari et al., 2020; Somers et al., 2020). The risk of this was found to be increased by use of Tocilizumab (IL-6 blocker) in ventilated COVID-19 patients from 26 to 54% of patients, despite apparent overall benefit of the treatment (Somers et al., 2020). Of interest, a recent study also showed that Type III interferon (lambda) is increased in the lower airways of COVID-19 patients and in a mouse model this was associated with damage to epithelial barriers and fatal bacterial superinfection (Broggi et al., 2020). This effect of Type III IFN is involved in severe IAV infection as well as revealed in a companion study, showing high levels of Type III IFN late in infection compromised epithelial repair in mice infected with IAV. These studies imply that timing of administration of IFNs or cytokine blockers may be key to achieve recovery from, or reduce incidence of, severe IAV or COVID-19 and that close attention should be paid to risks of bacterial superinfection.

Reactive oxygen and nitrogen species in part produced by myeloid cells have been strongly implicated in lung injury by IAV in many studies (Akaike et al., 1996; Snelgrove et al., 2006; Vlahos et al., 2011). For instance, mice lacking functional neutrophil oxidase has improved outcome with IAV infection. The anti-oxidant protein, NRF2, has been shown to play an important role in reducing cell injury during IAV infection (Kosmider et al., 2012). Blockade of reactive oxygen species with scavenger EUK-207 reduced lung damage and increased survival in mice infected with 1918 H1N1 (Kash et al., 2014).

Neutrophil extracellular traps (NETs) have recently been intensely studied in relation to their positive or negative roles in various infections of inflammatory states (Jenne and Kubes, 2015). NET formation is often linked to oxidant production by neutrophils; however, there are other potential pathways of NET formation (Boeltz et al., 2019). IAV elicits NET formation *in vitro* and *in vivo* (Narasaraju et al., 2011; Tripathi et al., 2014). It is possible may come into play more during severe IAV infection (e.g., they are induced by mechanical ventilation; Yildiz et al., 2015) It is in the setting of ARDS related to IAV that NETs appear to be playing a harmful pro-inflammatory role. Recent findings suggest a role for NETs in severe COVID-19 (Barnes et al., 2020; Zuo et al., 2020). Various mechanisms to block NET formation are under investigation. Histones are major components of NETs and they have been shown to mediate severe lung injury in a variety of settings, including in mouse models of IAV (Hoeksema et al., 2016; Ashar et al., 2018). Histones trigger profound inflammatory responses in the lung and lung injury and also trigger thrombotic events so they may provide a link between inflammatory injury and thrombosis in COVID 19 (Hoeksema et al., 2016; Pulavendran et al., 2019). We have recently shown that free histone H4, which has been found in lung lavage of patients with severe lung inflammation, directly activates neutrophil responses including the respiratory burst, degranulation and cytokine production (Hsieh et al., 2020). The mechanism for this activation involves membrane permeabilization and calcium influx into the cell. A similar phenomenon has been described for endothelial cells (Abrams et al., 2013a,b) and smooth muscle cells (Silvestre-Roig et al., 2019). This neutrophil activation by histones could be part of a feedback loop generating more inflammation.

A very important paper by et al. provides a potential explanation for conflicting data on the role of myeloid cells in severe IAV infection (Brandes et al., 2013). They performed a systems analysis of milder or more severe IAV infection in mice and showed that a feed forward circuit can develop in severe, lethal infection which is driven by neutrophils and related cytokines leading to inflammatory injury and death. Whereas, full ablation of all neutrophils was found to compromise outcomes in studies by Tate et al. (2011a), Brandes et al. performed more limited inhibition of neutrophil recruitment through low dose anti-neutrophil mAb treatment or reduction of HIF signaling and showed improved outcomes without increase in viral loads. HIF signaling was noted as a key driver of myeloid driven inflammation. These results suggest that a partial or modulated downregulation of neutrophil or myeloid activity may be an effective approach. Of interest they found that it was failure of early control of viral replication that led to development of the excessive neutrophilic inflammation. It appears the early neutrophilic response to IAV infection may be beneficial but that a late excessive neutrophil response may be harmful. This again implies that timing of interventions to down regulate neutrophil or other myeloid responses may be critical. In addition, as noted above paradoxical suppression of neutrophil responses by IFNs is associated with bacterial superinfection in IAV which must be taken into account in treatment with IFNs.

### Role of Soluble Airway Proteins in Host Defense Against IAV

We have studied a variety of innate soluble inhibitors of IAV most notably, the collectins and antimicrobial peptides. There are a variety of IAV inhibitors in respiratory lining fluid, including SP-D, SP-A, MBL, H-ficolin, LL-37, and other anti-microbial peptides, as we have recently reviewed (Tripathi et al., 2015c; Hsieh and Hartshorn, 2016; Hsieh et al., 2018). SP-D, SP-A, and H-ficolin are constitutively present in bronchoalveolar lavage fluid. In mice, SP-D levels were shown to increase in response to IAV infection, whereas SP-A did not (LeVine et al., 2001, 2002). LL-37 is mainly expressed in the lung during infection or inflammation (Gaudreault and Gosselin, 2008; Barlow et al., 2011) and is released both by respiratory and other epithelial cells and phagocytes. SP-D and MBL have been shown to bind to high mannose oligosaccharides on the seasonal viral hemagglutinin (HA) and reduce viral uptake into epithelial cells (Anders et al., 1990; Hartley et al., 1992; Hartshorn et al., 1994; Reading et al., 1997). Other inhibitors, including SP-A, H-ficolin, pentraxins, gp-340, and mucins contain sialic acid rich attachments on their surface to which the viral HA can bind limiting the ability of the virus to reach and attach to cellular sialic acid receptors (Iwaarden et al., 1993; Hartshorn et al., 1994, 2003, 2006a; Reading et al., 2008; Verma et al., 2012; Job et al., 2013). LL-37 appears to work by permeabilizing the viral membrane and blocking replication at an early stage of the viral life cycle (Tripathi et al., 2013). LL-37 expression is controlled by Vitamin D and can up increased by some HDAC inhibitors (Mily et al., 2015).

In mouse models, SP-D and SP-A reduce IAV replication *in vivo* while also limiting inflammatory responses (Reading et al., 1997; LeVine et al., 2001, 2002; Li et al., 2002; Hawgood et al., 2004; Vigerust et al., 2007). LL-37 has similar effects *in vitro* (Tripathi et al., 2013) and *in vivo* (Barlow et al., 2011). The IAV neutralizing activity of SP-D exceeds that of LL-37, SP-A, or Ficolins (e.g., for SP-D 50% inhibition occurs at ≈10 ng/ml vs. ≈10 μg/ml for SP-A or Ficolins). In murine studies absence of SP-D has a greater impact on viral replication and inflammation than absence of SP-A (LeVine et al., 2001, 2002; Li et al., 2002; Hawgood et al., 2004).

#### Role and Mechanism of Action of SP-D in Host Defense Against IAV

Human BALF from healthy donors has strong inhibitory activity for seasonal strains, and removal of SP-D significantly reduces this activity (Hartshorn et al., 1994; White et al., 2007). Of note, incubation of activated neutrophils with BALF leads to degradation of SP-D from the BALF (White et al., 2007). The degree of pathogenicity of various human IAV strains in mice correlates well with the extent of glycosylation on the viral HA. In particular, pandemic strains which have minimal HA glycosylation are much more pathogenic in mice than more heavily glycosylated seasonal strains (Vigerust et al., 2007; Hartshorn et al., 2008; Job et al., 2010; Tate et al., 2011b). Recombinant IAV strains containing HAs from 1918, 1957, 1968, or 2009 combined with the other genes from a seasonal strain, caused significantly greater morbidity and lower respiratory tract pathology in mice than a strain than the intact seasonal H1N1 strain (Qi et al., 2011). The increased pathogenicity of these strains correlated with a lack of ability of SP-D to bind to or inhibit these strains. An important limitation of the action of human or rodent SP-D vs. IAV is that it depends on presence high mannose oligosaccharides relatively near the sialic acid binding site of the HA. H1, and H3 that cause seasonal epidemics have evolved over time within the human populations to express more glycans on the binding head of their HA and as a result are more strongly inhibited by SP-D or MBL (Hartshorn et al., 1993; Vigerust et al., 2007; Tate et al., 2011b). It has been shown that such glycans protect this critical region of the HA from recognition by antiviral antibodies.

A very interesting recent paper evaluated the changes in glycosylation on the head region of the HA of human IAV strains including H1, H2, and H3 (Altman et al., 2019). The authors found that initial pandemic strains have few glycan attachments but glycans were added to the HA head region of H1 and H3 viruses at ~5–6 year intervals up to a maximum number of ~6–7 glycans. After this glycan positions are swapped at a slower rate. Addition of new glycans correlates with immune escape and dominance of the new strain which is not recognized by prior HA specific antibodies. The authors theorize that there are limits imposed on the addition of glycans in part due to the tendency of glycans to reduce receptor binding affinity. In addition, addition of high mannose glycans in particular increases susceptibility to inhibition by host defense lectins. Of interest, the H2 HA (which was not inhibited by SP-D; Hartshorn et al., 2008) was more limited in its ability to add glycans without major loss of binding and fusion activity of the HA and had a shorter period of circulation in humans than H1 or H3 viruses. Overall it seems likely that the resistance of human pandemic and avian strains to SP-D may be one factor in their pathogenicity for humans, although it is clear that other properties of the HA or other genes are involved in pathogenicity as well. As noted above other genes of the 1918 HIN1 viruses contribute to pathogenicity alongside the HA. In addition, as noted above some avian HAs confer strong pathogenicity in mice and others do not, and none of them are inhibited by SP-D (Qi et al., 2014).

**Figure 3:**
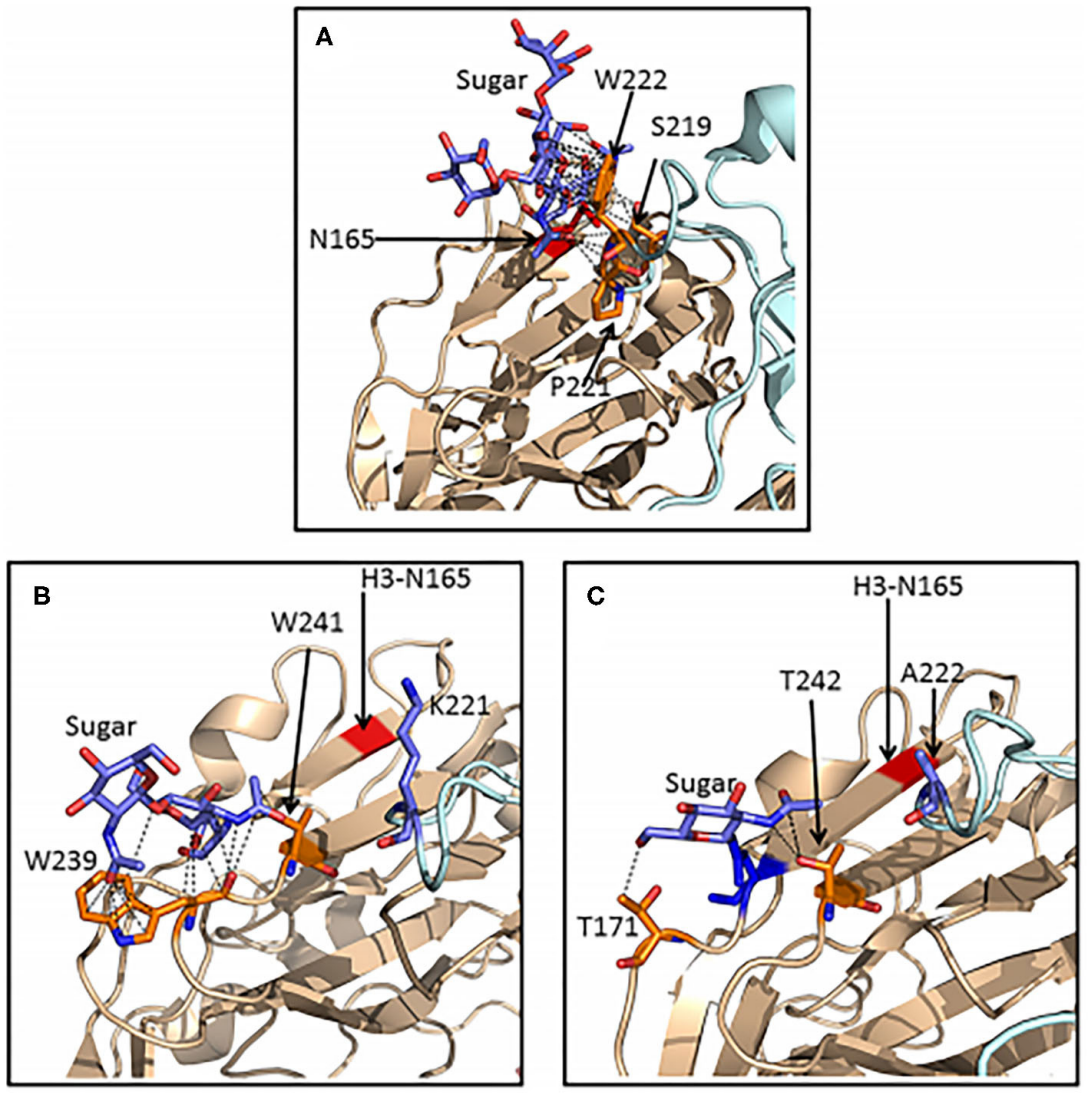
Ribbon representations of H3, H2, and H6 hemagglutinins showing extensive van der Waals contacts for the 165 glycan on H3 HA but not for glycans found on H2 or H6 HA. Crystal structures of H3 **(A)**, H2 **(B)**, and H6 **(C)** HA molecules were compared. Van der Waals contacts between the glycans and neighboring amino acids are shown as black dashed lines. Separate HA subunits are in wheat and pale cyan. The red stripe in panels B and C indicates the position of the key glycan for SP-D attachment at N165 in H3 hemagglutinin. The reduced amount of Van der Waals contacts for glycans found on H2 and H6 HA allows for processing by Golgi and ER enzymes such that the resulting glycan is complex rather than high mannose and hence not susceptible for binding by SP-D. This explains failure of SP-D to bind or inhibit these strains. This figure was obtained and adapted (Parsons et al., 2020) with permission allowed with proper citation.

Important advances in understanding the detailed mechanism of viral inhibition by SP-D has come from mass spectroscopy analysis of HA glycans, X ray crystallography and molecular modeling (Crouch et al., 2011; Goh et al., 2013; Khatri et al., 2016). All H3 subtype strains, including the 1968 pandemic strains, contain a high mannose glycan at position 165 on the HA head. This glycan is particularly well situated to allow binding of SP-D and blockade of the sialic acid binding site of the virus (Goh et al., 2013). Of interest, a glycan in a similar position is present in several avian HAs, including H2, H5, H6, and H11 avian HAs. However, these glycans are of complex type and do not allow for binding of SP-D (Parsons et al., 2020). Human and an avian H3 containing virus did allow binding of SP-D. Importantly, it was shown that differences in amino acid sequence and structure of the H2 and H6 HAs resulted in far fewer contacts between the N165 glycan and the protein surface than is seen with the H3 HA (Figure 3). The glycan on H2 or H6 avian strains are more accessible to Golgi and ER glycan processing enzymes explaining why they are of complex type in contrast to that on H3. This suggests that comparison of structures of various HAs could be used to predict whether high mannose glycans will be present and in turn whether SP-D or related lectins can bind or inhibit them.

SP-D is composed of trimers with an N-terminus, collagen domain, a neck and a carbohydrate recognition domain. These trimers further assemble into in highly multimerized forms with from 4 to 32 trimers in one molecule. These multimers are very potent in viral neutralization because of cooperativity of binding to viral glycans and an ability to also crosslink viral particles and cause viral aggregation (White et al., 2001, 2008; Tecle et al., 2008). This in turn promotes phagocyte uptake of the virus and limits uptake by epithelial cells. Presumably the aggregates are more susceptible to clearance through mucociliary processes as well.

Porcine SP-D is of great interest since it has increased and expanded activity antiviral activity compared with human or rodent SP-D (van Eijk et al., 2002, 2003, 2018). It is unique among SP-D types in having an N-linked glycan on its CRD which is highly sialylated and serves as a decoy ligand to which the viral HA can bind. This feature provides a mechanism for binding non-glycosylated IAV strains (van Eijk et al., 2018). In addition molecular features of the CRD head of porcine SP-D has several important changes compared to human and rodent SP-D that independently confer increased antiviral activity (van Eijk et al., 2012). Both of these features allow porcine SP-D to inhibit pandemic and even avian viral strains. Porcine SP-D also forms highly multimerized molecules as does human SP-D.

#### LL-37 and Other AMPs

LL-37 and defensins inhibit a broad spectrum inhibition of IAV strains, other viruses and bacteria (Gwyer Findlay et al., 2013; Hsieh and Hartshorn, 2016). We and others have shown that modified versions of LL-37, including D-isomers which are less susceptible to proteolysis, have increased activity vs. IAV (Tripathi et al., 2015a). Of interest, despite the fact that the antiviral activity of LL-37 does not depend on viral HA glycosylation, it did not inhibit strains from the 2009 H1N1 pandemic. However, a modified version was able to mediate inhibition (Tripathi et al., 2015a).

Defensins are another major class of host defense peptides and they are also produced by neutrophils and epithelial cells and have strong inhibitory activities for IAV. Unlike LL-37 they can induce viral aggregation and promote neutrophil and monocyte uptake of IAV (Tecle et al., 2007; Doss et al., 2009). Retrocyclins are cyclic antimicrobial peptides found in some primate species but not in humans. Synthetic versions of these cyclic peptides in natural or modified from have very strong IAV neutralizing and aggregating activity (Doss et al., 2009, 2012).

### Interaction of SP-D and Antimicrobial Peptides With Myeloid Cells

SP-D and other collectins modify responses to neutrophils, monocytes and macrophages to IAV or other stimuli. SP-D and MBL promote phagocytosis of IAV by human neutrophils and monocytes *in vitro* and increases neutrophil respiratory burst responses to the virus (Hartshorn et al., 1993, 1994; White et al., 2017). In the absence of other ligands collectins bind to macrophages through SIRPalpha and down-regulate activation of these cells, presumably contributing to an anti-inflammatory environment in the lung (Gardai et al., 2003). In addition, collectins can promote clearance of apoptotic cells by macrophages and SP-D knockout mice have an aberrant accumulation of apoptotic macrophages in the lung (Korfhagen et al., 1998; Palaniyar et al., 2003). Overall, in the resting state at least, SP-D and SP-A appear to assist in maintaining and anti-inflammatory milieu in the lung.

As noted above, IAV causes neutrophil dysfunction *in vitro* and *in vivo* and this could be a contributory factor to bacterial superinfection (Hartshorn et al., 1988, 1995; Abramson and Hudnor, 1994). SP-D and other collectins were able to reduce neutrophil dysfunction caused by the virus (Hartshorn et al., 1996). The ability of SP-D to increase viral uptake and respiratory burst responses is linked to its ability to cause viral aggregation and in turn to multimerization of the SP-D molecule (White et al., 2008). However, modified versions of SP-D containing only the trimeric NCRD are able to cause aggregation and increase viral uptake (Hartshorn et al., 2010). This property is based on the distinctive method of binding of the modified trimers to glycans on the viral HA (Goh et al., 2013). Of note, SP-D has been shown to bind to NETs which could in part explain its anti-inflammatory effects (Douda et al., 2011; Tripathi et al., 2015a).

LL-37 and other antimicrobial peptides also are able to modulate immune responses in various ways and have ability to promote responses of neutrophils and monocyte uptake of IAV and to modulate other phagocyte responses (Hartshorn et al., 2006b; Tecle et al., 2007; Doss et al., 2009, 2012; Tripathi et al., 2014, 2015b; Hsieh and Hartshorn, 2016). There are distinctions between the effects of defensins (and retrocyclins) and LL-37. Human neutrophil defensins and retrocyclins promote neutrophil uptake of IAV and bacteria but do not increase respiratory burst responses (Tecle et al., 2007; Doss et al., 2009). In contrast, LL-37 increases respiratory burst responses and NET formation by IAV, but does not cause viral aggregation or increase uptake of the virus by neutrophils (Tripathi et al., 2014, 2015b). The ability of LL-37 to potentiate IAV induced neutrophil respiratory burst responses is mediated through binding to formyl peptide type 2 receptors (Tripathi et al., 2015b). Note, however, that *in vitro* LL-37 downregulates IAV–induced monocyte inflammatory cytokine production (White et al., 2017) and *in vivo* it also downregulates cytokines during IAV infection *in vivo* (Barlow et al., 2011). Defensins have also been found to be anti-inflammatory (Miles et al., 2009; Semple et al., 2010).

Of interest, SP-D binds to defensins and retrocyclins, but not to LL-37 (Hartshorn et al., 2006b; Tripathi et al., 2013). As noted, addition of activated neutrophils to human BAL fluid reduces the amount of SP-D present and also reduces intrinsic antiviral activity of the fluid. This could be mediated either by proteases like elastase which are known to degrade SP-D or binding of SP-D to defensins released by the neutrophils. The ability of SP-D to bind to NETs may in part be mediated by binding to defensins and histones which are present in NETs.

Our findings regarding effects of antimicrobial peptides and collectins derive largely from *in vitro* studies with human neutrophils and monocytes. Further studies will be needed to determine which of these effects predominate *in vivo*, since some of them (e.g., potentiation of respiratory burst responses to IAV by LL-37 or SP-D) could be adverse but the net effect of SP-D or LL-37 *in vivo* is to reduce inflammation during IAV or other infections *in vivo* (Hawgood et al., 2004; Atochina-Vasserman et al., 2007; George et al., 2007; Ikegami et al., 2007; Hortobagyi et al., 2008; Jain et al., 2008; Barlow et al., 2011; Qi et al., 2011; Currie et al., 2016).

### Harnessing Innate Protein Inhibitors of IAV for Treatment

With regard to SP-D our laboratory has focused mainly on modifying the lectin binding property of the carbohydrate recognition domain to increase antiviral activity through two methods. Our first method was to replace the neck and carbohydrate recognition domain (NCRD) of SP-D with that of MBL or bovine conglutinin both of which have intrinsically stronger viral binding and inhibition capacity than human SP-Ds NCRD. These created as full length molecules and indeed had increased viral neutralizing activity for seasonal IAV strains and were able to restore the host defense function of SP-D when over-expressed in SP-D knockout mice (Hartshorn et al., 2000; White et al., 2000; Zhang et al., 2002). We next explored effectiveness of isolated trimers of the NCRD of SP-D. Using crystallographic data and modeling based on serum collectins (MBL, conglutinin or CL43), we were able to increase viral mannose binding affinity and viral neutralizing activity of these trimers and ultimately to demonstrate inhibition of pandemic strains not inhibited by wild type human or rodent SP-D (Nikolaidis et al., 2014; Hsieh et al., 2018). We were able to determine the molecular mechanisms through which the amino acid changes around the lectin binding site of SP-D conferred increased viral binding and neutralizing activity (Goh et al., 2013; Hsieh et al., 2018). Cross-linking of NCRD trimers by various means further increased activity (Hartshorn et al., 2010; White et al., 2010). While our modified NCRD proteins could inhibit both the 2009 and 1918 H1N1 strains, activity against these strains was ~10-fold less than activity against seasonal IAV or a 1968 H3N2 strain. In addition strains with no glycosylation on the NCRD (e.g., mouse adapted PR-8) or avian strains containing only complex glycans on the HA were not inhibited (Parsons et al., 2020).

**Figure 4:**
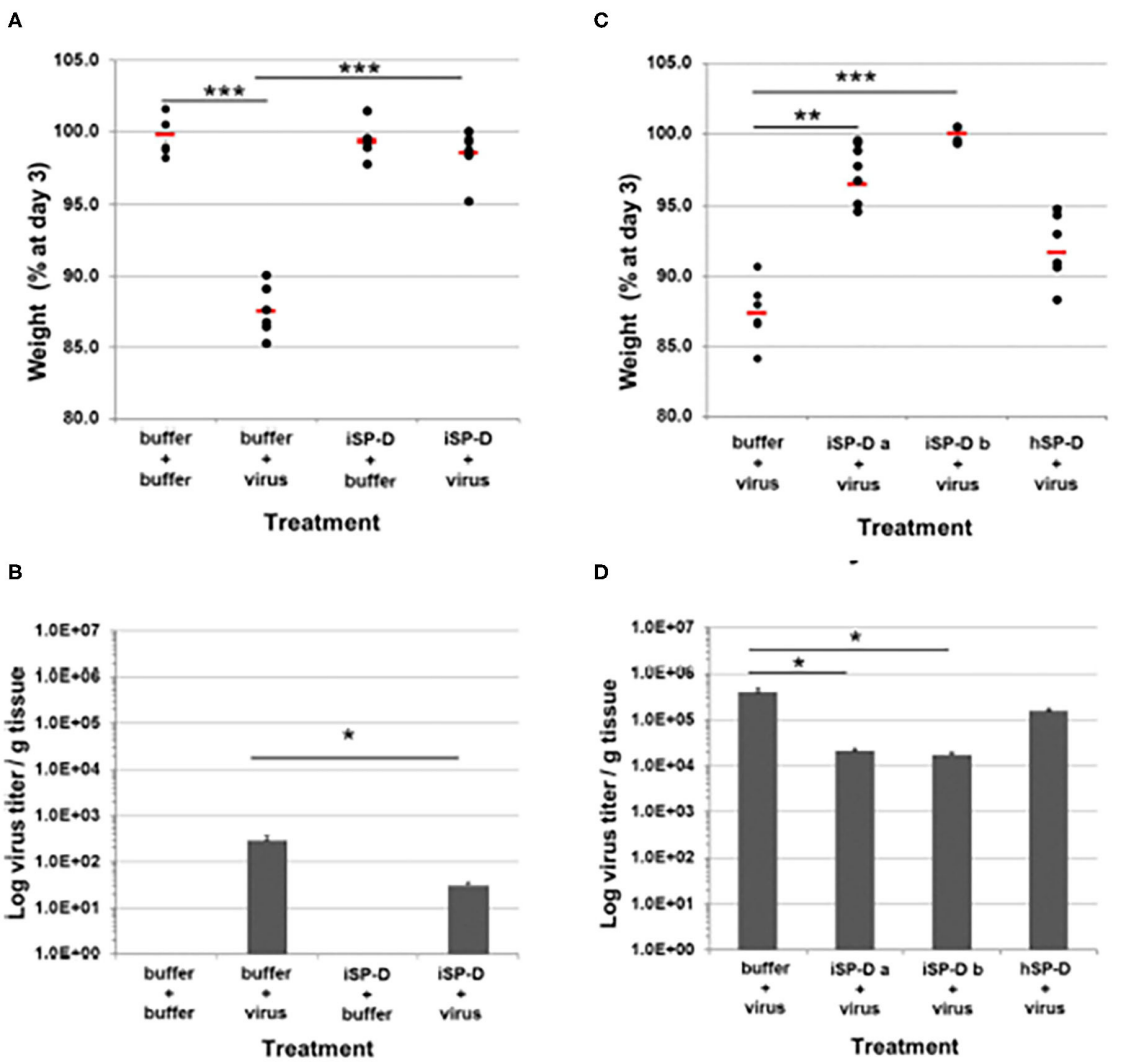
Validation of porcinized human SP-D as an antiviral agent against pandemic IAV infection in mice. The protective potential of porcinized human SP-D (iSP-D) against pandemic IAV infection was demonstrated *in vivo* (mouse model). Three different experiments were executed with 4 different conditions within each experiment; group size was *n* = 6 per condition. The A/California/E9/09 (H1N1) strain was inoculated along with either iSP-D or wild type human SP-D (hSP-D). Results in **(A,B)** show weights and virus titers, respectively for mice treated with PBS alone, Virus alone, iSP-D alone or iSP-D plus virus. **(B,C)** Show similar effects of two batches of iSP-D as compared to hSP-D. iSP-D provided significant protective effects which were greater than those provided by hSP-D which does not significantly inhibit this strain of virus *in vitro*. Statistical comparisons between experimental groups were made by Student's two-tailed paired *t*-test. *p < 0.05; **p < 0.005; ***p < 0.0005. This figure was obtained and adapted from (van Eijk et al., 2019) with permission allowed with proper citation.

As noted above porcine SP-D is a very promising platform for development of viral therapeutics. It has the advantage of dual mechanism of action resulting from the presence of an N-linked highly sialylated glycan on it CRD, and the enhanced glycan binding affinity of its lectin binding site. Recently Drs. Martin van Eijk and Henk Haagsman at University of Utrecht produced a version of human SP-D containing modifications derived from the NCRD of porcine SP-D in a full length, multimerized protein which was able to inhibit both avian and pandemic strains and the PR-8 strain even in the absence of the N-linked glycan present in porcine SP-D (van Eijk et al., 2019). This “porcinized” human SP-D reduced weight loss and viral titers when given to mice 24 h after infection in mice infected with pandemic IAV (Figure 4). Of further interest, when this SP-D was given 24 h before viral infection protected mice from weight loss without having a significant effect on viral load (as contrasted to given the SP-D 24 h after virus in which case viral titers and viral load are reduced). Adding the N-linked glycan to this version of SP-D may make it an ideal agent to both inhibit a broad spectrum of IAV strains and reduce excessive inflammatory responses. Of interest, another approach has been taken of creating chimeras of MBL with Ficolins which showed increased antiviral activities as well (Michelow et al., 2010; Takahashi et al., 2013).

In collaboration with Dr. Guangshun Wang (University of Nebraska School of Medicine) we showed that a modified version of LL-37 had increased IAV neutralizing activity and was able to inhibit pandemic IAV (Tripathi et al., 2015a). Many other labs are also working on methods to produce modified cathelicidins derived from LL-37 or cathelicidins from other species. As noted LL-37 (and some defensins) have the attractive property of both inhibiting IAV (and other viruses and bacteria, like SP-D), but also dampening inflammatory responses to IAV or other stimuli (Li et al., 2009; Miles et al., 2009; Barlow et al., 2011; Gwyer Findlay et al., 2013). We have also found that a range of novel defensins, some derived from primate retrocyclins not expressed in humans (Doss et al., 2009), and some totally novel (Doss et al., 2012), have increased activity vs. IAV. Such studies suggest that if these antimicrobial peptides can be safely administered they may be future tools for use in IAV or other severe respiratory viruses.

### Lessons From Research on Severe IAV Infection for Treatment of COVID-19

There are many similarities in manifestations of severe IAV and severe SAR-CoV infection, including profound, uncontrolled inflammatory response, massive cytokine release, diffuse alveolar damage, and lung infiltration with neutrophils (Table 1). It is likely that further studies will likely show major differences as well between severe IAV and severe SARS-CoV2 infection. One clear contrast is the striking role of thrombosis and endothelial dysfunction in COVID-19 which appears to exceed that in IAV as evidenced by the recent study by Ackermann et al. contrasting findings of lung pathology from fatal cases of COVID-19 and IAV (Ackermann et al., 2020). At first it seemed that bacterial superinfection might be less common in COVID-19 than IAV, but recent evidence summarized above, and the similar roles of IFNs in both illnesses, suggest that this may not be a major difference. Corticosteroids have not been rigorously studied in severe IAV but currently are not recommended. However, a recent clinical trial demonstrated an ~30% reduction in mortality of COVID-19 patients on ventilator support (Mahase, 2020). No benefit was seen in subjects not requiring oxygen support. However, it appears likely that the extensive body of data on immunopathogenesis of severe IAV will yield insights into severe COVID-19 illness and vice versa. More information is needed about the molecular mechanisms of COVID-19 lung and vascular injury to determine which mechanisms are shared with severe IAV. Multiple trials of immune-modulatory treatments and anti-coagulation are underway in COVID-19 and if positive could suggest potential trials to be done in severe IAV.

It appears likely that myeloid cells will be found to contribute to inflammatory damage in COVID-19 as they appear to do in severe IAV infection. Studies similar to those of Brandes et al. using targeted reduction of myeloid or neutrophil activity can be evaluated in animal models of COVID-19 as well (Brandes et al., 2013). A topic of special interest to us in this regard it the potential role of histones which are a major component of NETs and have been shown to contribute to lung injury in many setting and histone H4 can directly activate neutrophils and monocytes to generate pro-inflammatory cytokines and reactive oxygen species (Hsieh et al., 2020). Various proteins block inflammatory effects of histones including CRP, thrombomodulin, heparin, and anti-histone antibodies, with beneficial effects in mouse models (Hoeksema et al., 2016). We propose that further studies of neutrophil oxidants and NET components (including histones) will be valuable in COVID-19. These approaches may provide more targeted ways to interrupt the inflammatory cascade of severe IAV or COVID 19.

Like the HA of IAV and gp140 of HIV, the coronavirus spike protein is heavily glycosylated (Vankadari and Wilce, 2020). Thus, collectins could participate in host defense against SAR-CoV2. Prior studies have shown that SP-D and MBL can bind to and inhibit SARS-CoV1 and that gene polymorphisms of MBL which result in low serum levels of the protein are increased among SARS-CoV1 patients compared to controls (Ip et al., 2005; Zhang et al., 2005; Leth-Larsen et al., 2007). Hence, modified versions of SP-D could be of use in treatment of SAR-CoV2 as well. A lot more research needs to be done to understand the nature and role of glycosylation on the coronavirus spike protein and how it differs among strains or evolves in the human population. Important caveats include that SP-D and MBL have greatly reduced antiviral activity vs. HIV than IAV (Meschi et al., 2005). In addition, in some studies MBL and SP-D were found to potentiate rather than reduce Ebolavirus infection *in vitro* (Michelow et al., 2011; Favier et al., 2018). Hence, basic lab research of interaction of collectins with SARS-CoV2 will be the first starting point.

It is very likely also that antimicrobial peptides including LL-37 or defensins modified to reduce proteolysis and increase antiviral activity will have activity against SARS-CoV2. Hence, it will be important to test modified versions of these *in vitro*. Other strategies like increasing endogenous production of LL-37 with Vitamin D or HDAC inhibitors can be considered as well. A challenge for treatment with SP-D or peptides will be how to achieve adequate lung concentrations to inhibit the virus and possibly down-regulate inflammatory responses.

## Concluding Remarks

There is a vast literature on the pathogenesis of severe IAV infection and how it relates to innate immunity for better or worse. Remarkably, there is likely still a lot to learn and important new findings continue to appear regularly. The focus of this review is predominantly on the role of myeloid cells and soluble inhibitors of IAV including collectins like SP-D and antimicrobial peptides since these are the areas of our greatest expertise. Unfortunately it is very likely that IAV both in seasonal epidemic and in pandemic form will continue to be a major threat to human health and one can hope lesions learned from the COVID-19 pandemic will inform future responses to IAV infection as well. Methods of blocking inflammasome activation or neutrophil infiltration are being considered for COVID-19 and severe IAV, but as summarized above the literature suggests down-modulation rather than total blockade may be most beneficial. In general it will be important to balance the host defense role of elements of the innate response vs. both virus and potential superinfecting bacteria against their damaging effects. Timing of interventions may also be key as evidenced by the likely beneficial role of early IFN and neutrophil responses vs. their damaging effects when in excess at later times during the course of illness.

## Author Contributions

KH conceived and wrote the whole article but the work also summarizes results of a wide range of collaborators and that of other laboratories.

## Conflict of Interest

The author declares that the research was conducted in the absence of any commercial or financial relationships that could be construed as a potential conflict of interest.
